# An Internet-Based Means of Monitoring Quality of Life in Post-Prostate Radiation Treatment: A Prospective Cohort Study

**DOI:** 10.2196/resprot.3974

**Published:** 2015-09-28

**Authors:** Brent Parker, Rasika Rajapakshe, Andrew Moldovan, Cynthia Araujo, Juanita Crook

**Affiliations:** ^1^ British Columbia Cancer Agency Sindi Ahluwalia Hawkins Centre for the Southern Interior Kelowna, BC Canada; ^2^ Faculty of Medicine Department of Surgery University of British Columbia Vancouver, BC Canada; ^3^ Department of Computer Science (Unit 5) University of British Columbia, Okanagan Campus Kelowna, BC Canada; ^4^ Department of Statistics (Unit 5) University of British Columbia, Okanagan Campus Kelowna, BC Canada

**Keywords:** prostate cancer, radiation oncology, quality of life, patient-reported outcomes, Internet survey, prospective study

## Abstract

**Background:**

Widespread integration of the Internet has resulted in an increase in the feasibility of using Web-based technologies as a means of communicating with patients. It may be possible to develop secure and standardized systems that facilitate Internet-based patient-reported outcomes which could be used to improve patient care.

**Objective:**

This study investigates patient interest in participating in an online post-treatment disease outcomes and quality of life monitoring program developed specifically for patients who have received radiation treatment for prostate cancer at a regional oncology center.

**Methods:**

Patients treated for prostate cancer between 2007 and 2011 (N=1113) at the British Columbia Cancer Agency, Centre for the Southern Interior were invited by mail to participate in a standardized questionnaire related to their post-treatment health. Overall participation rates were calculated. In addition, demographics, access to broadband Internet services, and treatment modalities were compared between participants and nonparticipants.

**Results:**

Of the 1030 eligible invitees, 358 (358/1030, 34.7%) completed the online questionnaire. Participation rates were higher in individuals younger than age 60 when compared to those age 60 or older (42% vs 31%) and also for those living in urban areas compared with rural (37% vs 29%) and in those who received brachytherapy versus external beam radiotherapy (EBRT) (41% vs 31%). Better participation rates were seen in individuals who had access to Internet connectivity based on the different types of broadband services (DSL 35% for those with DSL connectivity vs 29% for those without DSL connectivity; cable 35% vs 32%; wireless 38% vs 26%). After adjusting for age, the model indicates that lack of access to wireless broadband connectivity, living in a rural area, and receiving EBRT were significant predictors of lower participation.

**Conclusions:**

This study demonstrates that participation rates vary in patient populations within the interior region of British Columbia, especially with older patients, those in rural areas, and those with limited access to quality Internet services.

## Introduction

As technologies improve and Canadians become more comfortable using computers and other Internet devices, there is an increasing potential to use online platforms as a means of communicating with patients on health-related issues. This communication allows for patient-reported outcomes (PROs), patient perspectives on their health and health care experience, to be collected. PROs are increasingly used as a valuable part of understanding the impact that an intervention has on a patient’s outcome. Therefore, these types of systems are progressively used to identify significant treatment-related morbidities and possess the potential for expansion in the field of clinical monitoring and quality of life (QOL) research [1-3]. Such systems also have potential to be implemented as a standard clinical process for specific health intervention programs; they could be beneficial in a number of areas, such as minimizing physician workload in routine patient follow-up consultations. Internet-based collection of PROs reduce travel time for patients requiring long-term follow-up, especially relevant for rural patients seeking specialized care [2]. Most importantly, the integration of PROs in clinical practice would allow for a more accurate reflection of patient health status, providing essential information about symptom management following treatment [1,2,4].

For this study, an Internet-based platform was designed for patient follow-up at the British Columbian Cancer Agency, Centre for the Southern Interior (BCCA-CSI), and a group of prostate cancer treatment patients treated with radiation therapy were invited to participate in an online disease-specific and QOL questionnaire. Both short- and long-term function and QOL are affected in men treated for prostate cancer, with declines in most functional domains occurring over at least 15 years of follow-up [5,6]. Therefore, future research in this area should assess both treatment efficacy and side effects so as to optimize treatment decisions and increase patient satisfaction and QOL following cancer treatment [6]. In addition, the use of PROs in this patient population could enable oncologists to identify patients or patient types that may benefit from improved postradiation therapy management.

The outcomes of interest in this study were twofold: the overall participation rate of an online PRO system implemented as a pilot and the participation rates based on readily available personal, demographic, and treatment-related factors. The overall goal of the initiative was to evaluate feasibility of a regional oncology program to eventually transition to Web-based collection of PROs following cancer treatment, so as to further inform patient-centered care and better understand the long-term impacts of cancer treatments.

##  Methods

### Platform Design

An in-house online platform was designed to host participant data and collect the online participant response information in an automated format; it pulls information from completed surveys from an external server to a secure database and dashboard. The project was approved by the Institutional Ethics Review Board as a quality improvement project and the online platform was reviewed for institutional privacy impact and met the necessary data security standards of practice.

### Patient Selection and Recruitment

The BCCA is an agency responsible for province-wide, population-based oncologic care and radiation therapy for British Columbians undergoing treatment through 6 regional cancer centers including CSI. Patients with nonmetastatic prostate cancer treated with conventional radiation therapy are discharged to their primary care practitioner within 2 years of treatment and are often not seen again unless re-referred back to the program.

In January 2014, all living individuals (N=1113) treated with radiation therapy for prostate cancer between 2007 and 2011 at the BCCA-CSI were invited by mail to complete a standardized questionnaire related to their post-treatment health. Therefore, all patients were at least 2 years out of their initial treatment during the course of this study.

These men were mailed a single letter (see Multimedia Appendix 1), which provided a description of the study, statement of their right to accept or decline participation, study code that enabled them to log into the secure online platform, and instructions on how to consent and complete the questionnaire through the online platform. Invitees were also encouraged to call the study coordinator (BP) if they had questions related to the study or questionnaire. The questionnaire consisted of questions on the urinary function, rectal toxicity, and sexual health components of the Expanded Prostate Cancer Index Composite, a validated and commonly used set of functional and QOL surveys for prostate cancer patients [7]. Individuals were given 4 months to complete the online questionnaire. They were provided with no other form of communication or invitation to participate.

### Statistical Analysis

Spatial analysis was performed in ArcGIS (Esri), which is a spatial visualization and analysis software program. For the spatial analysis, each patient’s address was converted into a geographic coordinate. Broadband Internet connectivity data were obtained from the 2012 Broadband Canada: Connecting Rural Canadians’ National Broadband Maps derived from an initiative completed by Industry Canada [8]. Broadband Canada defined the presence of broadband services as a minimum download speed of 1.5 megabits per second. Three different types of geographical broadband data were available: cable, digital subscriber line (DSL), and wireless (ground based and satellite based). Subsequently, the broadband Internet connectivity data were joined to each geocoded coordinate based on linear proximity to the nearest broadband geographic coordinate data point. In addition, spatial statistical analysis calculated the distance of each patient from the nearest of the 5 provincial radiation treatment facilities. These distances were grouped into the following 3 categories: less than 200 km, 200-400 km, and more than 400 km. Furthermore, a rural/urban status was established for each invitee based on his postal code.

Statistical analysis was performed in SPSS version 14 (IBM Corp). A chi-square, *t* test, or the nonparametric equivalent was used to compare participation rates by patient characteristics. The relationship between covariates of interest and participation was initially calculated through univariate binary logistic modeling. Subsequent analysis indicated that many of the covariates were associated with each other. Therefore, an age-adjusted model was developed, resulting in an age-adjusted model and odds ratio estimates for each covariate of interest. All tests of statistical significance were two sided and the threshold for statistical significance was set at *P*<.05.

##  Results

### Patient Demographics and Participation Rates

Of the 1113 individuals invited to participate in the study, 83 were subsequently excluded due to mortality near time of letter mail out or otherwise lost to follow-up due to reasons such as a change of address. The characteristics of participants and nonparticipants are described in Table 1. Overall, 358 (358/1030, 34.7%) of the 1030 invitees completed the online questionnaire. Participation rates were higher in individuals younger than 60 years compared with those aged 60 years and older (139/334, 41.6%, vs 219/696, 31.5%). Similarly, participation rates were higher in urban areas, when compared with rural (262/700, 37.5%, vs 96/330, 29.1%), and for those who received brachytherapy versus external beam radiotherapy (EBRT; 156/380, 41.1%, vs 202/650, 31.1%). Participation was also greater from individuals who had access to Internet connectivity based on the types of broadband (DSL 337/958, 35.1%, for those with DSL connectivity vs 21/72, 29.2%, for those without DSL connectivity; cable 316/898, 35.2%, for those with cable connectivity vs 42/132, 31.8%, for those without cable connectivity; wireless 290/770, 37.7%, for those with wireless connectivity vs 68/260, 26.2%, for those without wireless connectivity).

### Predictors for Participation

Both the univariate and age-adjusted model odds ratios and their statistical significance for these measures are reported in Table 2. After adjusting for age, the model indicates that rural status, lack of access to wireless broadband connectivity, and prior EBRT treatment remain as significant predictors of relatively lower participation.

**Table 1:** Demographic factors of participants and nonparticipants.

		Participated (N=358)	Invited, but did not participate (N=672)	*P* value
Age, years, at time of letter mail out, mean (SD)		73 (8)	72 (7)	.001
Rural area of residence, n (%)		96 (27)	234 (35)	.009
**Residence distance from center, km, n (%)**				
	<200	295 (82)	542 (81)	.495
	200-400	41 (11)	75 (11)	—
	>400	22 (6)	55 (8)	—
**Types of broadband connectivity, n (%)**				
	DSL	337 (94)	621 (92)	.369
	Cable	316 (88)	582 (86)	.494
	Wireless	290 (81)	480 (71)	.001
**Primary radiation treatment type, n (%)**				
	EBRT	202 (56)	448 (67)	.001
	BT	156 (44)	224 (33)	—

**Table 2:** Predictors for participation, represented in crude and age-adjusted odds ratios.

	Crude model			Age-adjusted model		
	Odds ratio	*P* value	95% CI	Adjusted odds ratio	*P* value	95% CI
Age (each year increasing)	0.973	.001	0.957-0.990	—	—	—
Urban versus rural (reference: urban)	0.686	.009	0.517-0.910	0.701	.014	0.528-0.931
Distance from center (increasing)	0.893	.324	0.714-1.118	0.915	.439	0.730-1.147
DSL connectivity (reference: yes)	0.759	.303	0.449-1.283	0.78	.357	0.461-1.322
Cable connectivity (reference: yes)	0.859	.448	0.581-1.271	0.856	.439	0.578-1.268
Wireless connectivity (reference: yes)	0.586	.001	0.429-0.801	0.601	.001	0.439-0.823
Radiation treatment type (reference: EBRT)	1.545	.001	1.187-2.010	1.39	.021	1.052-1.837

##  Discussion

### Principal Results

The results of this study show modest participation rates; even for individuals under the age of 60, only 40% completed the survey. However, individuals were made aware of the study exclusively by a single mailed letter; reminders may have increased participation. Informing patients about this PRO aspect of care and follow-up in the time surrounding their treatment and integrating email invitations and reminders would also likely increase participation rates; however, integration of these response improvement strategies was beyond the scope of the pilot project.

Previous studies including patients with prostate and other cancers have indicated that it may be feasible to use Web-based technologies as a means of collecting PROs and follow-up [9-11]. Vickers and colleagues [10] reported a 39% participation rate for all eligible postprostatectomy patients (age range 57-65 years). Sebrow et al [9] demonstrated a 50% participation rate in prostatectomy patients (age range 38-77 years). The latter group suggested that these types of systems could be useful for collection of post-treatment QOL, especially from those patients who may not otherwise attend follow-up due lack of geographic proximity to a treatment facility. Although both of these previously reported studies had higher participation rates, they also had a younger population, different method of recruitment, and briefer interval between treatment and invitation to participate when compared to this study. As expected, participation rates were higher in individuals younger than 60 years compared to those aged 60 years and older. Internet usage is strongly dependent on user attitude, especially in areas such as perceived ease of use and perceived access barriers [12,13]. With regard to age, Porter et al [13] noted that older individuals (aged 50 years and older) typically exhibit lower perceived ease of use, in addition to perceiving more access barriers associated with Internet usage. Future action may require the development of educational tools and support to familiarize patients with the online platforms and help reduce user anxiety. In addition, it was seen that likelihood of participation rates was higher in brachytherapy patients. In contrast to patients treated with conventional radiotherapy, brachytherapy patients are never discharged but remain on regular follow-up for at least 10 years. They are accustomed to filling out the questionnaires used in this study, and their follow-up data are recorded in a provincial database operational since 1998. Furthermore, at our clinic it has been observed that brachytherapy patients are generally more involved in shared decision making and are often more self-educated than other patients. These behaviors may be associated with an increased likelihood of responding to a questionnaire invitation.

As expected, participation rates were significantly lower in rural areas and in areas without wireless broadband connectivity. The majority (96%) of the regions without wireless broadband had either DSL broadband or cable broadband. Wireless broadband connectivity requires wireless transmitters, the same technology that supplies wireless Internet to cellular phones. While online platforms have allowed for more widespread collection of PROs in certain instances, they are also prone to excluding certain populations due to access and use of Internet [14,15]. As access to wireless broadband was a significant predictor of participation in this study, it is hypothesized that those who have limited access to cellular mobile and smartphone Internet connectivity may also be less likely to use other Web-based technologies. Rural addresses were also associated with lower participation and, as the rural regions of the BC southern interior are often forested and mountainous, residents are likely to have limited and unreliable access to wireless connectivity. Furthermore, there may also be cultural differences regarding general Internet usage in rural regions compared to urban regions.

These findings are applicable and relevant to other Web-based health surveillance, health monitoring and research programs currently in development. Missing data and sample bias are two of the most serious, practical problems involved with implementing online PRO platforms. As health care institutions increasingly employ modern Web-based technologies in patient management, it will be important to monitor the uptake of these technologies by elderly patients and those living in rural areas [1,15] and develop strategies to increase uptake and minimize health disparities within these patient cohorts.

### Limitations

This study has several limitations. The mailing information for the BCCA-CSI was outdated, and 55 (5%) invitation letters were returned due to an invalid address. In addition, the broadband connectivity data used within the spatial analysis was based on data published in 2012 and therefore may not be fully representative of BC’s current broadband coverage.

### Conclusions

These findings demonstrate that a modest proportion of prostate cancer patients treated at the BCCA-CSI are willing to use online systems to report health outcomes. Our results demonstrate that participation rates vary based on age, geographic location, and access to certain types of Internet connectivity. As specialist care increasingly uses Web-based technologies to interact with patients and monitor their health as part of standard post-treatment and long-term clinical monitoring, usage of these technologies in rural residents and older patients should be monitored to ensure that these patient cohorts continue to receive appropriate medical care.

## Appendix

Multimedia Appendix 1 Web follow-up letter of contact.

## Abbreviations

BCCA-CSI: British Columbian Cancer Agency, Centre for the Southern Interior
DSL: digital subscriber line
EBRT: external beam radiotherapy
PRO: patient-reported outcomes
QOL: quality of life
