# Supplementary material for: An Internet-Based Means of Monitoring Quality of Life in Post-Prostate Radiation Treatment: A Prospective Cohort Study
Source: JMIR Res Protoc. 2015 Sep 28;4(3):e115. doi: 10.2196/resprot.3974 (PMC4704929; doi:10.2196/resprot.3974)
Supplement: Multimedia Appendix 1 [file resprot_v4i3e115_app1.pdf]

BC Cancer Agency
CARE & RESEARCH
An agency of the Provincial Health Services Authority
SOUTHERN INTERIOR

Dear Mr. _____________,

You are invited to participate in a research study investigating **Web Follow-up for Men Treated for Prostate Cancer at the BC Cancer Agency – Centre for the Southern Interior**. An internet based questionnaire which allows Oncologists to continue to monitor health and side effects related to treatment has been developed. This study will investigate if internet-based questionnaires are a practical way to deliver post-treatment follow-up.

To participate, please follow the directions below to enter the web-site and fill in two short surveys on-line. These surveys do NOT replace any regular follow-up that is scheduled with your oncologist or regular medical doctor.

To participate in this study:

1. Go onto http://www.MyFollowup.ca/
2. Click Participate and enter this code:__________________
    a. This code ensures that your information is kept confidential
    b. Only the approved doctors and medical and research staff at the BCCA-SAHCSI will have access to this information
3. Read and accept the consent form
4. Complete the two listed surveys.
    a. This should take 5-10 minutes to complete
    b. The surveys will ask several multiple choice questions related to your urinary, rectal and sexual health
5. Click the log-out button to finish
6. You will be invited to complete this survey every six months for two years

Thank you for considering to participate in this study, and if you have general questions related to it, please feel free to contact:

- The Study Coordinator: Brent Parker, Telephone: 250-712-3900 ext 686878
- The Principal Investigator: Dr. Rasika Rajapakshe, Telephone: 250-712-3915

Sincerely,

Juanita Crook

Dr. Juanita Crook, MD, FRCPC
Radiation Oncologist
BC Cancer Agency - Centre for the Southern Interior
Kelowna, BC
